# The Role of Body Mass Index (BMI) in Differentiated Thyroid Cancer: A Potential Prognostic Factor?

**DOI:** 10.3390/biomedicines12091962

**Published:** 2024-08-29

**Authors:** Chiara Mele, Lucrezia De Marchi, Giulia Marsan, Marco Zavattaro, Maria Grazia Mauri, Paolo Aluffi Valletti, Gianluca Aimaretti, Paolo Marzullo

**Affiliations:** 1 Department of Translational Medicine, University of Piemonte Orientale, 28100 Novara, Italy; 20022109@studenti.uniupo.it (G.M.); gianluca.aimaretti@med.uniupo.it (G.A.); paolo.marzullo@med.uniupo.it (P.M.); 2 Department of Endocrinology, UZ Brussel, Laarbeeklaan, 1090 Brussels, Belgium; lucredem@gmail.com; 3 Division of Endocrinology, University Hospital “Maggiore della Carità”, 28100 Novara, Italy; marco.zavattaro@med.uniupo.it (M.Z.); grazia.mauri@maggioreosp.novara.it (M.G.M.); 4 ENT Division, University of Piemonte Orientale, 28100 Novara, Italy; paolo.aluffi@med.uniupo.it

**Keywords:** thyroid cancer, body mass index, obesity, tumor aggressiveness, outcome

## Abstract

Obesity has been recognized as a potential risk factor for the carcinogenesis of differentiated thyroid cancer (DTC). The aim of this observational study was to investigate the prognostic role of BMI in influencing DTC histopathological aggressiveness and the risk of tumor relapse. We enrolled 257 patients with DTC, consecutively admitted to our Institution between January 2016 and December 2023. The following variables were collected: demographic, anthropometric and clinical parameters, risk factors for DTC, surgical and radioiodine therapy, histopathological features of DTC, and biochemical markers of disease. Tumor recurrence was assessed during short-, medium- and long-term follow-up. According to BMI tertiles (e.g; I: BMI < 23.3 kg/m^2^; II: 23.3 ≤ BMI < 27.1 kg/m^2^; III: BMI ≥ 27.1 kg/m^2^), the clinical and histopathological characteristics did not differ between groups. The multinomial logistic regression analysis showed that BMI was not associated with clinical and histopathological aggressiveness of DTC, independently from sex, age, and risk factors for DTC onset. Moreover, BMI did not constitute a predictor of tumor recurrence during follow-up. In conclusion, BMI does not represent a predictor of clinical and histopathological aggressiveness of DTC. Since it is not a reliable marker of adiposity, BMI cannot be considered alone in evaluating the potential association between obesity and DTC prognosis.

## 1. Introduction

Obesity represents a potential risk factor for many types of cancer [1,2], and body mass index (BMI) has been found to be associated with histological and clinical aggressiveness of prostate and breast cancer [3]. Over the last decades, a concomitant increase in the incidence of obesity and differentiated thyroid cancer (DTC) has been documented worldwide [4,5]. While the epidemiological trend of DTC can be partly attributed to a greater diagnostic accuracy [6,7,8], an increased incidence of larger tumors with aggressive histological features has led some authors to speculate on the increasing exposure to specific risk factors, including radiation, environmental pollutants, and nutritional factors [9,10,11].

Recently, a slight but significant association was found between the incidence of DTC and the growing prevalence of obesity, suggesting a possible role of obesity in thyroid carcinogenesis [12,13].

Although the mechanisms relating obesity and DTC remain largely unknown, the potential impact of insulin resistance and proinflammatory adipocytokines as risks factors for thyroid tumorigenesis has been postulated [14,15,16].

Furthermore, several authors have investigated the potential role of obesity as a risk factor for the aggressiveness of thyroid cancer, with conflicting results. Some studies have reported overweight/obesity as a predictor of aggressive histopathological features of DTC (i.e., larger tumor size, multifocality, extrathyroidal invasion, and lymph node metastases) [17,18,19,20], while others did not find any relationship between obesity and DTC histopathological aggressiveness [21,22,23,24].

Conflicting results have also been published about the risk of DTC relapse in patients with obesity. Some authors observed that patients with obesity have an increased risk of developing locoregional recurrence of papillary thyroid cancer (PTC) during long-term follow-up [25], although most of the studies did not demonstrate a higher risk of tumor relapse in patients with obesity as compared to normal-weight subjects [21,22,24].

Given the lack of univocal results on the relationship between obesity and DTC histology and outcome, the aim of our study was to investigate the potential prognostic role of BMI in influencing DTC histopathological aggressiveness and risk of tumor relapse.

## 2. Materials and Methods

### 2.1. Study Design and Population

This observational, retrospective, cohort study included 257 patients with DTC, consecutively admitted to the Endocrinology Unit of the University Hospital “Maggiore della Carità” of Novara, between January 2016 and December 2023. Patients with a history of poorly differentiated, anaplastic, or medullary thyroid carcinomas were excluded.

The study design conformed to the ethical guidelines of the Declaration of Helsinki and was approved by the local Ethical Committee “Comitato Etico Territoriale Interaziendale AOU Maggiore della Carità di Novara”. All of the participants signed a written informed consent form.

### 2.2. Variables, Data, Sources and Measurements

Data were retrieved from electronic hospital records and included the following variables: demographic and anthropometric characteristics, known risk factors for DTC, clinical and family history, surgical approach, histopathological features of DTC, AJCC VIII ed.’s TNM and staging classifications [26], radioiodine (RAI) therapy, and tumor recurrence at 1, 3, and 5 years from diagnosis. Recurrence was defined in accordance with the 2015 ATA dynamic response system [27]. The patients were followed up at regular intervals with periodical biochemical and ultrasonographic evaluations, and timing of follow-up was based on the dynamic risk re-stratification process [27,28]. Thyroglobulin (Tg) levels were considered to evaluate the clinical response when thyroglobulin antibodies (TgAb) were negative. Contrariwise, in the presence of interfering TgAb, their values were considered to define the response to treatment [29].

#### 2.2.1. Body Measurements

All subjects underwent body measurements wearing light underwear, in fasting conditions after voiding, as described previously [15]. Weight and height were measured in the nearest 0.1 kg and 0.1 cm, respectively, using standard methods. BMI was expressed as body mass (kg)/height (m)^2^. Overweight was defined as any BMI between 25 and 29.9 kg/m^2^ and obesity as any BMI over 30 kg/m^2^.

#### 2.2.2. Laboratory Tests

Serum Tg and TgAb were measured with the same methods in all patients at the baseline and during each follow-up visit. Serum Tg levels were determined using an automated chemiluminescence method (LIAISON XL, DiaSorin S.p.A, Saluggia, Italy), with a functional sensitivity of 0.1 ng/mL.

Plasma levels of TgAb were assessed using an automated chemiluminescence assay system (Anti-Tg Ready Pack, Siemens Healthcare Diagnostics). TgAb positivity was defined as serum TgAb levels above 60 IU/mL.

#### 2.2.3. Neck Ultrasound (US)

The US was performed with an Esaote MyLab Twice real-time US system with a linear multifrequency (7–14 MHz) probe. Central and laterocervical lymph node compartments were inspected during each follow-up visit to assess the presence of a locoregional recurrence of the disease. Suspicious lymph nodes were evaluated by using US-guided fine-needle aspiration cytology (FNAC) in association with the Tg measurement in washing fluid.

#### 2.2.4. Surgery

Surgery was performed by the same experienced thyroid surgeon, so that the thyroidectomy and lymph node dissection techniques were consistent across patients. The choice of the type of surgery and lymph node dissection was collegially made by the surgeons and endocrinologists according to ATA guidelines recommendations [27].

#### 2.2.5. Thyroid Histology

Two independent pathologists reviewed the histological slides for the following assessments: tumor size, histological type, foci number, (multi)focality, extrathyroidal extension, vascular invasion, surgical margins, tumor-associated thyroiditis, and presence of loco-regional and/or distant metastases, according to the 8th edition of the AJCC TNM Staging System [26].

#### 2.2.6. Radioiodine Therapy (RAI)

RAI was performed after withdrawal of levothyroxine (L-T4) or after recombinant human thyrotropin stimulation (rhTSH). The ^131^I uptake was measured using a 2-head gamma camera (e.cam Dual-Head 180° Gamma Camera Siemens Medical System, Erlangen, Germany). The choice to perform RAI was made according to the ATA guidelines and recent evidence [27,30,31]

### 2.3. Statistical Analysis

Values are expressed as mean ± standard deviation (SD) or absolute number and percentage. Data points not normally distributed, obtained by the Shapiro–Wilk test, were log-transformed to improve the symmetry and homoscedasticity of the distribution. The univariate ANOVA for continuous variables and the chi-square tests for categorical variables were used for comparisons between the subgroups of BMI tertiles. Multinomial logistic regression analysis was used to evaluate the association between BMI as a continuous variable and the clinical-histopathological features of DTC, after controlling for age, sex, and risk factors for DTC occurrence.

Univariate logistic regression analysis was conducted to test the potential association between tumor relapse and clinical-histopathological features of DTC. Multivariable logistic regression analysis was performed to identify the independent risk factors of tumor relapse. The regression models included the individual variables considered in the univariate analysis and potential confounders, including sex, age, BMI, histological type, radiation exposure, thyroiditis, and smoking habit. The odds ratio (OR), 95% confidence interval (95% CI), and related significant values obtained from regression are reported. Statistical significance was set at 5%. Statistical analyses were performed using SPSS version 21 (Somers, NY, USA).

## 3. Results

### 3.1. Clinical and Histopathological Characteristics

A summary of the clinical and histopathological characteristics of the study population is reported in Table 1. Most patients were female (70.4%, male-to-female ratio 1:2.4), with a mean age at diagnosis of 53.8 ± 15.2 years (range 17–88 years). The majority of patients were Caucasian (93.4%). The mean BMI was 25.9 ± 5.2 kg/m^2^, and overweight was found in 87 cases (33.9%) and obesity in 45 cases (17.5%). Smoking habits were documented in 18.7% of cases. Regarding the risk factors for thyroid cancer, 16 patients (8.5%) were exposed to radiation in the head and neck region, and 55 patients (21.4%) had autoimmune thyroiditis. Overall, 15.9% of patients had a history of cancer in a different site; moreover, the most frequent types were breast cancer in women (29.3%), prostate cancer in men (12.2%), and lymphoma in both sexes (19.5%). A family history of thyroid nodules, autoimmune thyroiditis, and thyroid cancer was found in 11.3%, 4.7%, and 5.4% of cases, respectively. Familiarity for other malignancies was found in 24.5% of patients.

Most of the patients underwent total thyroidectomy (94.2%), with concomitant prophylactic or therapeutic dissection of the central compartment in 30.0% and 37.4% of cases, respectively. In patients with US suspicion of lateral neck lymphadenopathy, a dissection of the lateral neck compartment was also performed (12.1% of cases).

The most frequently found histotype was PTC (87.1%) and the most common variant was the follicular one (39.20%), followed by the classical one (37.9%). The average tumor size was 16.6 ± 13.1 mm, with tumors < 10 mm (microcarcinomas) in 36.2% of cases. Multifocality and bilaterality were detected in 94 (36.6%) and 66 (25.7%) cases, respectively. Extrathyroidal extension was detected in 57 patients (22.3%) and vascular invasion in 43 patients (16.7%).

In more than a quarter of patients (28.9%), lymph node metastases were documented, prevalently in the central compartment (59.4%). The average number of involved lymph nodes was 6.3 ± 8.6.

Based on these histopathological features, and according to the 8th edition of the AJCC TNM Staging System, overall, 65.4% of cases were classified as stage I, followed by stage III (18.3%), stage II (9.3%), and stage IV (7.0%).

Overall, 100 patients (38.9%) underwent radioiodine (RAI) treatment within 6 months after surgery. The average dose of 131I was 81.5 ± 23.2 mCi. The mean stimulated Tg level was 14.5 ± 45.5 ng/mL, and a minority of patients showed TgAb positivity (12.0%). The majority of patients (84.0%) showed whole-body scintigraphy (WBS) uptake in the thyroid bed, while an uptake in the lateral neck compartment or in multiple sites was detected in the 3.0% and 12.0% of cases, respectively (Table 2).

**Figure 1:**
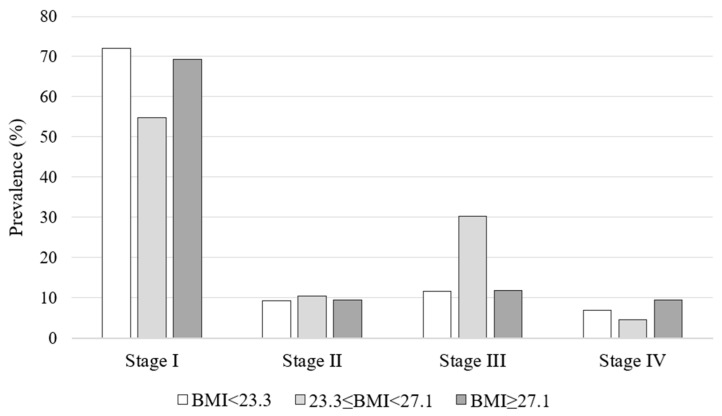
Stage of disease according to BMI tertiles.

When the population was subgrouped according to BMI tertiles (I tertile: BMI < 23.3 kg/m^2^, II tertile: 23.3 ≤ BMI < 27.1 kg/m^2^, BMI ≥ 27.1 kg/m^2^), no significant differences were found in terms of clinical and histopathological features (Table 1), stage of disease (Figure 1), and dosimetric, laboratory, and WBS characteristics in the context of RAI (Table 2).

A multinomial logistic regression analysis was conducted to evaluate the association between BMI as a continuous variable and the clinical-histopathological characteristics of the tumor (Table 3). BMI was not associated with clinical and histopathological features, independently from potential confounders, including sex, age, and risk factors for DTC.

### 3.2. Follow-Up

Tumor relapse was observed in 35 patients (15.7%) at one year after diagnosis, in 15 patients (10.5%) at 3 years after diagnosis, and in 12 patients (17.1%) at 5 years after diagnosis. Most of the patients had biochemical evidence of disease (87.1%), whereas, in only eight patients (12.9%), biochemical and instrumental evidence of disease was documented. Of the latter, five patients had local recurrence at the laterocervical lymph nodes, while three patients had lung metastases.

Data on short-term (1 year), medium-term (3 years), and long-term (5 years) follow-up, divided according to tertiles of BMI, are summarized in Table 4. No significant differences in tumor recurrence rates were found between the three groups of BMI tertiles.

### 3.3. Risk of Tumor Relapse

Univariate logistic regression analysis was conducted to test the potential risk factors of tumor relapse in our population. BMI and risk factors for DTC were not associated with an increased risk of relapse in the short-, medium-, and long-term follow-up. Among the clinical and histopathological features, extrathyroidal extension (OR = 2.29, CI 95% 1.24–4.22, *p* = 0.008), tumoral infiltration of surgical margins (OR = 2.44, CI 95% 1.05–5.69, *p* = 0.04), the presence of lymph node metastases at diagnosis (OR = 4.00, CI 95% 1.65–9.73, *p* = 0.002), and high stimulated Tg levels in the context of RAI (OR = 1.03, CI 95% 1.01–1.05, *p* = 0.007) were identified as risk factors for one-year tumor relapse.

With regard to medium- and long-term follow-up, tumoral infiltration of surgical margins (OR = 4.31, CI 95% 1.44–12.87, *p* = 0.009) and one-year tumor relapse (OR 11.79, CI 95% 3.20–43.48, *p* < 0.0001) represented independent predictors of three-year tumor relapse, while five-year tumor relapse was independently predicted only by one-year tumor relapse (OR = 20.8, CI 95% 3.29–131.91, *p* = 0.001).

Multivariable logistic regression analysis was subsequently conducted to better characterize the associations found with univariate analysis (Table 5). The regression models included the individual variables considered in the univariate analysis to avoid collinearity and potential confounders, including sex, age, BMI, histological type, radiation exposure, thyroiditis, and smoking habit. The analysis confirmed that the variables identified in the univariate analysis represent risk factors for DTC recurrence, regardless of the potential confounders mentioned above.

## 4. Discussion

The present study investigated the potential association between BMI and aggressiveness of DTC. Our results showed that BMI is not associated with histopathological aggressiveness of DTC, nor with a higher risk of tumor recurrence in the short-, medium-, and long-term follow-up.

Over the last decade, several studies have analyzed the potential relationship between obesity and DTC occurrence. A meta-analysis published in 2021 demonstrated that obesity is associated with a 50% increase in the risk of thyroid cancer (RR = 1.5, CI 95% 1.45–1.55) [32]. In addition, obesity has been claimed to play a role in influencing the histopathological and clinical aggressiveness of DTCs [17,18].

Different pathogenetic mechanisms have been proposed to define the association between obesity and the onset of DTC. An important role in thyroid carcinogenesis could be played by TSH, the levels of which are slightly increased in patients with obesity [33,34,35]. In this context, while some studies reported an association between higher TSH values and a greater risk of malignancy of thyroid nodules, as well as the aggressiveness of DTC [36,37], others failed to document significant differences in TSH values among DTC patients depending on the presence of obesity, thus downscaling the contribution of circulating TSH on susceptibility to thyroid malignancy in people with obesity [38,39]. Even the excess of adipose tissue seems to promote cancer cell growth and metastasis through a condition of insulin resistance and altered secretion of IGF-1, which have a direct stimulating action on thyrocyte growth [40]. In this setting, the production of adipocytokines by adipose tissue could also be involved in the relationship between obesity and thyroid cancer; moreover, in particular, increased leptin secretion seems to be associated with a poor prognosis and greater tumor aggressiveness [41,42]. On the other hand, adiponectin, which has an insulin-sensitizing and anti-inflammatory action, is reduced in obese subjects and is inversely correlated with BMI [15,43].

On this basis, several studies have evaluated the relationship between obesity and the aggressiveness of thyroid cancer, with controversial results. While some authors found no association between BMI and the histopathological characteristics of thyroid cancer [23,24], others identified a relationship between BMI and some aggressive features of DTC, including tumor size > 1 cm and extrathyroidal extension [19,44].

A recent study by Matrone et al. evaluated 1058 patients with DTC enrolled at the time of first RAI treatment and subgrouped them according to BMI categories (underweight, normal weight, overweight, and obesity) [24]. The authors did not find associations between the clinical-histopathological characteristic of DTC and BMI. However, this study has some limitations, which include the lack of information on risk factors for DTC (e.g., radiation exposure and history of chronic autoimmune thyroiditis) and a family history of thyroid neoplasms. A further limitation is that the authors collected the BMI values at the first RAI treatment, which is commonly performed 3 to 6 months after surgery, a period during which the BMI could be affected by inadequate thyroid compensation.

In the present study, we collected anthropometric and clinical features at the time of DTC diagnosis, in the context of euthyroidism or adequate thyroid compensation, thus avoiding any temporary changes in BMI values. Moreover, we considered potential confounders including family history and the presence of specific risk factors for DTC. No significant associations were observed between BMI and histopathological characteristics of tumor aggressiveness, such as histological type, size, bilaterality, multifocality, extrathyroidal extension, vascular invasion, and the presence of lymph node metastases at diagnosis, after controlling for sex, age at diagnosis, and risk factors for DTC. As opposed to our findings, Kim et al. observed that higher BMI values are associated with PTC size > 1 cm, the presence of extrathyroidal extension, and advanced TNM stage [19]. These results could be explained by the fact that the Asian population, compared to the Caucasian population, has a lower incidence of obesity. In fact, only 4.6% of patients included in the study of Kim et al. were obese, compared to 17.5% in our study and 19.9% in the study by Matrone et al.

A direct association between BMI and extrathyroidal extension was also observed in other studies [19,44], but these included heterogeneous cohorts and a clear prevalence of a single ATA risk class of DTC.

As regards RAI treatment, we did not find any differences in terms of dose of ^131^I administered, stimulated Tg levels, positivity of TgAb, and WBS results among the three subgroups of BMI tertiles. These results, again, seem to suggest that BMI per se cannot be considered as a marker of DTC aggressiveness.

Finally, we investigated the association between BMI and tumor recurrence in the short-, medium-, and long-term follow-up. In addition, in this case, BMI was not associated with high DTC aggressiveness in terms of biochemical and/or instrumental tumor recurrence. This has been previously reported in other studies [24,45]. In our population, the main predictors for short-term DTC recurrence were extrathyroidal extension, tumoral infiltration of surgical margins, the presence of lymph node metastases at diagnosis, and high stimulated Tg levels in the context of RAI, while medium- and long-term DTC recurrence were independently predicted by one-year tumor relapse.

Our study has several limitations that should be pointed out, as follows: first, the lack of information about the BMI changes that occurred after DTC diagnosis; second, the single-center nature of the study with a limited sample size, especially with regard to follow-up data; and third, the absence of indicators of body composition and fat distribution.

In conclusion, BMI does not represent a predictor of clinical and histopathological aggressiveness of DTC. Since BMI remains an arguable (surrogate) marker of adiposity, it cannot be considered alone in evaluating the potential association between obesity and DTC prognosis. Body composition parameters assessed via bioimpedance or total body DEXA could add key information for evaluating the role of obesity in thyroid cancer aggressiveness.

## Figures and Tables

**Table 1:** Clinical and histopathological characteristics of the population as a whole and subdivided into three groups according to BMI tertiles (1st tertile: BMI < 23.3 kg/m^2^; 2nd tertile: 23.3 ≤ BMI < 27.1 kg/m^2^; 3rd tertile: BMI ≥ 27.1 kg/m^2^).

Variables	Whole Population (N = 257)	BMI I Tertile (N = 86)	BMI II Tertile (N = 86)	BMI III Tertile (N = 85)	*p*-Value
Sex					
Male	76 (29.6)	16 (18.6)	34 (39.5)	26 (30.6)	0.01
Female	181 (70.4)	70 (81.4)	52 (60.5)	59 (69.4)	
Age at diagnosis	53.8 ± 15.2	49.5 ± 17.0	57.7 ± 14.5	54.1 ± 12.9	0.002
Ethnicity					
Caucasian	240 (93.4)	82 (95.3)	78 (90.7)	80 (94.1)	0.07
Hispanic	9 (3.5)	1 (1.2)	3 (3.5)	5 (5.9)	
Asian	5 (1.9)	3 (3.5)	2 (2.3)	0 (0.0)	
African	3 (1.2)	0 (0.0)	3 (3.5)	0 (0.0)	
Risk factor					
Radiation	16 (6.2)	6 (7.0)	5 (5.8)	5 (5.9)	0.94
Thyroiditis	55 (21.4)	21 (24.4)	15 (17.4)	19 (22.4)	0.52
Smoking	48 (18.7)	17 (19.8)	18 (20.9)	13 (15.3)	0.61
Other tumors	41 (15.9)	15 (17.4)	12 (14.0)	14 (16.5)	0.81
Family history					
Thyroid nodules	29 (11.3)	12 (14.0)	9 (10.5)	8 (9.4)	0.62
Thyroid cancer	14 (5.4)	5 (5.8)	3 (3.5)	6 (7.1)	0.58
Thyroiditis	12 (4.7)	4 (4.7)	3 (3.5)	5 (5.9)	0.76
Other tumors	63 (24.5)	23 (26.7)	19 (22.1)	21 (24.7)	0.78
Surgery					
Lobectomy	15 (5.8)	6 (7.0)	5 (5.8)	4 (4.7)	0.82
Total thyroidectomy	242 (94.2)	80 (93.0)	81 (94.2)	81 (95.3)	
pCND	77 (30.0)	23 (26.7)	30 (34.9)	24 (28.2)	0.46
tCND	96 (37.4)	37 (43.0)	29 (33.7)	30 (35.3)	0.40
LCD	31 (12.1)	14 (16.3)	7 (8.1)	10 (11.8)	0.26
Histotype					
PTC	224 (87.1)	77 (89.5)	74 (86.0)	73 (85.9)	0.96
FTC	22 (8.6)	6 (7.0)	8 (9.3)	8 (9.4)	
Hurtle Cells	11 (4.3)	3 (3.5)	4 (4.7)	4 (4.7)	
PTC variants					
Classical	81 (37.9)	28 (39.4)	31 (43.1)	22 (31.0)	0.67
Follicular	84 (39.2)	25 (35.2)	25 (34.7)	34 (47.9)	
Classical and follicular	28 (13.1)	10 (14.1)	10 (13.9)	8 (11.3)	
Other variants	21 (9.8)	8 (11.3)	6 (8.3)	7 (9.8)	
FTC variants					
Minimally invasive	14 (63.6)	3 (50.0)	5 (62.5)	6 (75.0)	0.63
Invasive	8 (36.4)	3 (50.0)	3 (37.5)	2 (25.0)	
Tumor size (mm)	16.6 ± 13.1	16.1 ± 12.1	17.3 ± 13.5	16.3 ± 14.0	0.82
Bilaterality	66 (25.7)	19 (22.1)	26 (30.2)	21 (24.7)	0.46
Multifocality	94 (36.6)	25 (29.1)	38 (44.2)	31 (36.5)	0.12
Extrathyroidal extension	57 (22.3)	21 (24.4)	19 (22.1)	17 (20.0)	0.78
Vascular invasion	43 (16.7)	14 (16.3)	10 (11.6)	19 (22.4)	0.17
Surgical margins					
R0	225 (87.5)	77 (89.5)	74 (86.0)	74 (87.1)	0.68
R1	31 (12.1)	9 (10.5)	11 (12.8)	11 (12.9)	
R2	1 (0.4)	0 (0.0)	1 (1.2)	0 (0.0)	
LND MTS at diagnosis					
Presence of LND MTS	59 (28.9)	22 (29.7)	20 (30.3)	17 (26.6)	0.88
N° of LND MTS	6.3 ± 8.6	7.8 ± 9.0	4.9 ± 8.5	6.1 ± 8.4	0.58
Location of LND MTS					
Central compartment	35 (59.4)	10 (45.5)	17 (85.0)	8 (47.1)	0.06
LC compartment	12 (20.3)	7 (31.8)	1 (5.0)	4 (23.5)	
Both	12 (20.3)	5 (22.7)	2 (10.0)	5 (29.4)	

Data are expressed as mean ± SD or absolute number and percentage. Comparison between groups was performed using univariate ANOVA or χ^2^ test. For 3-group comparison analysis, the statistical significance was set at 1% (*p* < 0.01). Abbreviations: pCND, prophylactic central neck dissection; tCND, therapeutic central neck dissection; LCD, laterocervical; PTC, papillary thyroid cancer; FTC, follicular thyroid cancer; LND, lymph nodes; MTS, metastases.

**Table 2:** Dosimetric, laboratory, and WBS characteristics of patients who underwent RAI therapy.

Variables	Whole Population (N = 100)	BMI I Tertile (N = 33)	BMI II Tertile (N = 34)	BMI III Tertile (N = 33)	*p*-Value
RAI activity (mCi)	81.5 ± 23.2	84.3 ± 26.2	82.2 ± 20.2	77.9 ± 23.2	0.52
Stimulation regimen					
LT4-withdrawal	82 (82.0)	29 (87.9)	31 (91.2)	22 (66.7)	0.02
rhTSH	18 (18.0)	4 (12.1)	3 (8.8)	11 (33.3)	
Stimulated Tg (ng/dL)	16.7 ± 40.6	26.8 ± 60.2	11.0 ± 23.0	13.6 ± 31.9	0.27
TgAb					
Positive (≥30 IU/mL)	12 (12.0)	4 (12.1)	3 (8.8)	5 (15.2)	0.73
Negative (<30 IU/mL)	88 (88.0)	29 (87.9)	31 (91.2)	28 (84.8)	
WBS uptake					
No uptake	0 (0.0)	0 (0.0)	0 (0.0)	0 (0.0)	0.60
Thyroid bed	84 (84.0)	28 (84.9)	29 (85.3)	27 (81.8)	
Neck LCC	3 (3.0)	0 (0.0)	2 (5.9)	1 (3.0)	
Mediastinum or lungs	0 (0.0)	0 (0.0)	0 (0.0)	0 (0.0)	
Bone	1 (1.0)	1 (3.0)	0 (0.0)	0 (0.0)	

Data are expressed as mean ± SD or absolute number and percentage. Comparison between groups was performed using univariate ANOVA or χ^2^ test. For 3-group comparison analysis, the statistical significance was set at 1% (*p* < 0.01). Abbreviations: RAI, radioiodine, LT4, levothyroxine; rhTSH, recombinant human thyrotropin; Tg, Thyroglobulin; TgAb, Thyroglobulin antibodies; LCC, laterocervical compartment; WBS, whole-body scintigraphy.

**Table 3:** Multinomial logistic regression analysis to evaluate the association between the BMI as a continuous variable and the histopathological features of the tumor.

Variables	BMI (Continuous Variable)			
	OR (CI 95%)	*p*-Value	OR (CI 95%) (Sex*Age*RFs)	*p*-Value (Sex*Age*RFs)
Histotype				
PTC	Ref	-	Ref	-
FTC	1.01 (0.93–1.10)	0.83	1.01 (0.92–1.11)	0.80
Hurtle Cells	0.99 (0.89–1.12)	0.98	0.99 (0.88–1.13)	0.97
PTC variants				
Classical	Ref	-	Ref	-
Follicular	1.06 (0.99–1.13)	0.07	1.07 (1.00–1.14)	0.05
Classical and follicular	1.03 (0.94–1.12)	0.55	1.04 (0.96–1.14)	0.34
Other variants	1.02 (0.90–1.16)	0.77	1.02 (0.88–1.17)	0.82
FTC variants				
Minimally invasive	Ref	-	Ref	-
Invasive	1.02 (0.85–1.22)	0.87	1.03 (0.84–1.25)	0.80
Tumor size				
≤1 cm	Ref	-	Ref	-
>1 cm but ≤2 cm	0.97 (0.92–1.03)	0.35	0.97 (0.92–1.03)	0.34
≥2 cm but ≤4 cm	0.98 (0.92–1.04)	0.48	0.98 (0.92–1.04)	0.50
>4 cm	1.04 (0.95–1.13)	0.44	1.03 (0.94–1.13)	0.56
Bilaterality				
No	Ref	-	Ref	-
Yes	1.02 (0.97–1.07)	0.53	1.02 (0.96–1.07)	0.55
Multifocality				
No	Ref	-	Ref	-
Yes	1.03 (0.98–1.08)	0.29	1.02 (0.97–1.08)	0.34
Extrathyroidal extension				
No	Ref	-	Ref	-
Yes	1.01 (0.95–1.07)	0.87	0.99 (0.94–1.06)	0.91
Vascular invasion				
No	Ref	-	Ref	-
Yes	1.05 (0.99–1.11)	0.14	1.05 (0.99–1.11)	0.12
Surgical margins				
R0	Ref	-	Ref	-
R1	1.03 (0.96–1.10)	0.49	1.02 (0.95–1.09)	0.52
R2	0.97 (0.65–1.17)	0.90	0.99 (0.70–1.43)	0.98
LND MTS at diagnosis				
No	Ref	-	Ref	-
Yes	1.01 (0.96–1.07)	0.65	1.02 (0.96–1.08)	0.62
Location of LND MTS				
Central compartment	Ref	-	Ref	-
LC compartment	1.04 (0.94–1.15)	0.47	1.03 (0.92–1.15)	0.60
Both	0.98 (0.87–1.10)	0.70	0.98 (0.86–1.11)	0.69

Abbreviations: OR, odds ratio; CI, confidence interval; RFs, risk factors; PTC, papillary thyroid cancer; FTC, follicular thyroid cancer; LND, lymph nodes; MTS, metastases; LC, laterocervical.

**Table 4:** Short-, medium-, and long-term follow-up in the total population and divided according to BMI tertiles.

Variables	Whole Population	BMI I Tertile	BMI II Tertile	BMI III Tertile	*p* Value
1-year tumor relapse					
No	188 (84.3)	62 (80.5)	65 (90.3)	61 (82.4)	0.10
Biochemical evidence of disease	32 (14.3)	12 (15.6)	7 (9.7)	13 (17.6)	
Biochemical and instrumental evidence of disease	3 (1.4)	3 (3.9)	0 (0.0)	0 (0.0)	
3-year tumor relapse					
No	128 (89.5)	46 (92.0)	39 (90.7)	43 (86.0)	0.90
Biochemical evidence of disease	11 (7.7)	3 (6.0)	3 (7.0)	5 (10.0)	
Biochemical and instrumental evidence of disease	4 (2.8)	1 (2.0)	1 (2.3)	2 (4.0)	
5-year tumor relapse					
No	58 (82.9)	24 (82.8)	15 (83.3)	19 (82.6)	0.52
Biochemical evidence of disease	11 (15.7)	5 (17.2)	2 (11.1)	4 (17.4)	
Biochemical and instrumental evidence of disease	1 (1.4)	0 (0.0)	1 (5.6)	0 (0.0)	

Data are expressed as absolute number and percentage. Comparison between groups was performed using χ^2^ test.

**Table 5:** Multivariable logistic regression analysis used to evaluate the risk factors for DTC recurrence in the short-, medium-, and long-term follow-up.

Variables	OR (CI 95%) Model Weighted for Sex, Age, BMI, RFs, and Histological Type	*p*-Value Model Weighted for Sex, Age, BMI, RFs, and Histological Type
1-year follow-up		
Extrathyroidal extension	2.62 (1.36–5.05)	0.004
Infiltration of surgical margins	2.80 (1.17–6.72)	0.02
LND MTS at diagnosis	5.20 (1.95–13.88)	0.001
Stimulated Tg level	1.03 (1.01–1.06)	0.01
3-year follow-up		
Infiltration of surgical margins	4.84 (1.52–15.43)	0.008
One-year tumor relapse	14.01 (3.46–56.74)	<0.0001
5-year follow-up		
One-year tumor relapse	32.29 (3.80–274.55)	0.001

Abbreviations: OR, odds ratio; CI, confidence interval; LND, lymph nodes; MTS, metastases; Tg, thyroglobulin.

## Data Availability

The data presented in this study are available upon request from the corresponding author due to privacy.
